# Assessing evidence of interventions addressing inequity among migrant populations: a two-stage systematic review

**DOI:** 10.1186/s12939-019-0970-x

**Published:** 2019-05-06

**Authors:** Jirawit Yadee, Mukdarut Bangpan, Kednapa Thavorn, Vivian Welch, Peter Tugwell, Nathorn Chaiyakunapruk

**Affiliations:** 1 Department of Pharmaceutical Care, Faculty of Pharmacy, Chiang Mai University, Chiang Mai, Thailand; 2 Evidence for Policy and Practice Information and Coordinating Centre (EPPI-Centre), UCL Institute of Education, University College London, London, UK; 3 Ottawa Hospital Research Institute, The Ottawa Hospital, Ottawa, Ontario Canada; 4 School of Epidemiology and Public Health, Faculty of Medicine, University of Ottawa, Ottawa, Ontario Canada; 5 Institute of Clinical and Evaluative Sciences, ICES uOttawa, Ottawa, Ontario Canada; 6 Bruyere Research Institute, University of Ottawa, Ottawa, Ontario Canada; 7 Department of Medicine, Faculty of Medicine, University of Ottawa, Ottawa, Ontario Canada; 8 Clinical Epidemiology Program, Ottawa Hospital Research Institute, Ottawa, Ontario Canada; 9 WHO Collaborating Centre for Knowledge Translation and Health Technology Assessment in Health Equity, Bruyère Research Institute, Ottawa, Ontario Canada; 10 School of Pharmacy, Monash University Malaysia, Jalan Lagoon Selatan, 46150 Bandar Sunway, Selangor Malaysia; 11 Center of Pharmaceutical Outcomes Research (CPOR), Department of Pharmacy Practice, Faculty of Pharmaceutical Sciences, Naresuan University, Phitsanulok, Thailand; 12 School of Pharmacy, University of Wisconsin, Madison, USA; 13 Asian Centre for Evidence Synthesis in Population, Implementation and Clinical Outcomes (PICO), Health and Well-being Cluster, Global Asia in the 21st Century (GA21) Platform, Monash University Malaysia, Bandar Sunway, Selangor Malaysia; 14 Department of Pharmacotherapy, College of Pharmacy, University of Utah, Salt Lake City, Utah USA

**Keywords:** Migrant, Health, Intervention, Equity

## Abstract

**Background:**

Everyone has the right to achieve the standard of health and well-being. Migrants are considered as vulnerable populations due to the lack of access to health services and financial protection in health. Several interventions have been developed to improve migrant population health, but little is known about whether these interventions have considered the issue of equity as part of their outcome measurement.

**Objective:**

To assess the evidence of health interventions in addressing inequity among migrants.

**Methods:**

We adopted a two-stage searching approach to ensure the feasibility of this review. First, reviews of interventions for migrants were searched from five databases: PubMed, Cochrane, CINAHL, PsycINFO, and EMBASE until June 2017. Second, full articles included in the identified reviews were retrieved. Primary studies included in the identified reviews were then evaluated as to whether they met the following criteria: experimental studies which include equity aspects as part of their outcome measurement, based on equity attributes defined by PROGRESS-Plus factors (place of residence, race/ethnicity, occupation, gender, religion, education, socio-economic status, social capital, and others). We analysed the information extracted from the selected articles based on the PRISMA-Equity guidelines and the PROGRESS-Plus factors.

**Results:**

Forty-nine reviews involving 1145 primary studies met the first-stage inclusion criteria. After exclusion of 764 studies, the remaining 381 experimental studies were assessed. Thirteen out of 381 experimental studies (3.41%) were found to include equity attributes as part of their outcome measurement. However, although some associations were found none of the included studies demonstrated the effect of the intervention on reducing inequity. All studies were conducted in high-income countries. The interventions included individual directed, community education and peer navigator-related interventions.

**Conclusions:**

Current evidence reveals that there is a paucity of studies assessing equity attributes of health interventions developed for migrant populations. This indicates that equity has not been receiving attention in these studies of migrant populations. More attention to equity-focused outcome assessment is needed to help policy-makers to consider all relevant outcomes for sound decision making concerning migrants.

**Electronic supplementary material:**

The online version of this article (10.1186/s12939-019-0970-x) contains supplementary material, which is available to authorized users.

## Introduction

Migrant populations often face health inequities which occur from inequalities in social and economic conditions [1] that impact on the risk of illness and the need for healthcare. In addition, migrants are often vulnerable due to lack of access to health services and financial protection in health [2]. According to the United Nations (UN) population division estimate, the number of international migrants has dramatically increased since 1960, rising from 77 million to almost 244 million in 2015, 3.3% of the world’s population [3].

According to the World Health Organization (WHO) Constitution of 1948, the right to achieve the standard of health and well-being belongs to everyone including migrants and refugees [4]. In 2016, The 17 Sustainable Development Goals (SDGs) of the 2030 Agenda for Sustainable Development officially came into force. This agenda affirmed the need for monitoring to ensure that no one is left behind. To reduce inequalities, health policies should contribute to the achievement of SDG 3 on ensuring healthy lives and promoting well-being for all, SDG 5 on achieving gender equality, and SDG 10.7 on reducing inequalities by facilitating orderly, safe, and responsible migration and mobility of people, including through implementation of planned and well-managed migration policies. It is important for government and health systems to consider these aspects, to understand the barriers to health, and to then initiate interventions and approaches to improve the health of disadvantaged populations [1, 5].

A number of reviews have reported the effects of interventions aimed at improving health in migrant populations [6–14] with clinically relevant outcomes. However, little is known about whether these interventions have considered the issue of equity and used relevant equity-focused outcomes as part of their assessment. In addition, the inequity issue is crucial for the health system and is considered as a part of the global strategy. Health interventions should not only improve clinical outcomes but should also address the social disparity issue which is one of the SDGs. This review assesses whether health interventions reduce health inequities within migrant populations or consider equity as part of their measurement among the migrant populations. Findings from our study are expected to improve the understanding of current health intervention studies targeted at migrants. The review is particularly important since migrant populations are considered in the context of achieving target 10 of the global strategy on reducing inequities.

## Methods

The review is reported according to the PRISMA-Equity 2012 Statement [15] (Preferred Reporting Items for Systematic Review and Meta-Analysis with a focus on health Equity). We defined migrant populations as any people who moved across an international border away from their original place of residence, regardless of the voluntariness of movement [16]. To gather the evidence on equity attributes addressed in this review, we used the PROGRESS-Plus framework by members of the Campbell and Cochrane Equity Methods group [17].

### Literature search

Since there have been a number of reviews on interventions developed to improve population health of migrant populations in the literature [6–14], we adopted a two-stage searching approach to ensure the feasibility of this review. First, a search was conducted in five databases (PubMed, Cochrane, CINAHL, PsycINFO, and EMBASE) from inception to June 2017. The main search terms for literature searches included “Migrant”, “Health” and “Review”. We adopted the search terms for “immigrant” and “systematic review”. Further details on search terms used in literature search are shown in Table 1. A detailed example of a full electronic search is placed in Additional file 1: Table S1. There was no language restriction in this systematic review. We searched for reviews in this first stage and then retrieved the full articles of the studies included in each review to check whether they met the inclusion criteria. Second, we screened primary studies identified from each review which met the criteria from the first stage.

**Table 1:** Search terms

	Search terms
Literature review	“data synthesis” OR “evidence synthesis” OR metasynthesis OR meta-synthesis OR “narrative synthesis” OR “qualitative synthesis” OR “quantitative synthesis” OR “realist synthesis” OR “research synthesis” OR “synthesis of evidence” OR “thematic synthesis” OR metaanaly* OR meta-analy* OR “scoping stud*” OR meta-ethnograph* OR meta-epidemiological OR “systematic review” OR “scoping review” OR “rapid review”
Migrant	refugees OR refugee OR refugee camps OR camp OR refugee OR camps OR aliens OR alien OR emigrants OR emigrant OR foreigners OR foreigner OR immigrants OR immigrant OR migrant OR migrants OR asylum-seekers OR “internally displaced person”
Health	Health

^*^ truncation operator represents zero or more terminal characters in a search term

### Inclusion criteria

Two stages of inclusion criteria were used. To be included in the first stage review, the review must meet the following inclusion criteria; [1] a review that reported the effect of health interventions [2] a review that included experimental studies comparing intervention(s) to standard/control group or before/after interventions [3] participants were migrants or immigrant, refugee, asylum seekers, or internally displaced persons. In the second stage, we included only primary studies with an experimental study design (randomized controlled trials or quasi-experimental studies) because these designs allow meaningful evaluation when equity is included as part of the outcome measurement of the health intervention. This is consistent with the goal of this review which is to determine whether evaluation of interventions have included equity attributes as part of their outcome measurement. The equity attributes of interest covered both social and economic risk factors using the PROGRESS-Plus categories (place of residence, race/ethnicity, occupation, gender, religion, education, socio-economic status, social capital, and others) as part of the outcome measurement. Further detail on the inclusion criteria of both stages is shown in Table 2.

**Table 2:** Inclusion criteria

Inclusion criteria for the first stage review	
Study design	Literature review that reported the effect of health interventions with or without health equity based on PROGRESS-Plus factors^a^ and subjected to a comparative evaluation (compared to standard/control group or before/after interventions)
Population	Participants are migrants^b^
Inclusion criteria for the second stage review	
Study design	Experimental studies (RCT or Quasi-experimental studies) that reported the effect of health interventions with health equity based on PROGRESS-Plus factors ^a^ 1) Studies reported the effect of intervention on reducing inequity according to PROGRESS-Plus^a^ 2) Studies reported the potential difference of the effect of intervention on outcomes based PROGRESS-Plus^a^
Population	Participants are migrants^b^

^a^Data on health equity: PROGRESS-Plus - Place of residence, Race/ethnicity/culture/language, Occupation, Gender/sex, Religion, Education, Socioeconomic status, Social capital and “Plus” to indicate other possible factors such as disease status or disability

^b^Including other terms of migrants: immigrant, refugee, asylum seekers, and internally displaced person

### Article screening and data extraction

One reviewer (JY) conducted the literature search, performed the screening and information extraction from the included studies. One reviewer (MB) verified the extracted data. Full texts of articles passing the second stage screening were retrieved, and their eligibility and quality assessed independently by two reviewers (NC and KT). Any discrepancy in the screening process was resolved through discussion. Two independent reviewers discussed the results. Extracted information included authors, year of study, study population, outcomes specified in the included studies, and findings related to PROGRESS-Plus factors as determinants of health equity.

### Quality assessment and data analysis

Quality assessment for randomized controlled trials was performed using the Revised Cochrane risk of bias tool for randomized trials (RoB) version 2.0 [18]. Quality assessment for quasi-experimental studies was guided by Risk Of Bias In Non-randomized Studies of Interventions (ROBINS-I) [19]. Since there was no quantitative data similar enough to be pooled across studies, we could not perform quantitative synthesis. It was also not possible to use the GRADE (Grading of Recommendations Assessment, Development and Evaluation) framework for consideration of health equity as the overall effect estimates and uncertainty could not be estimated in our study. We narratively summarized all findings using a content analysis approach [20, 21].

## Results

### Study selection

**Fig. 1:**
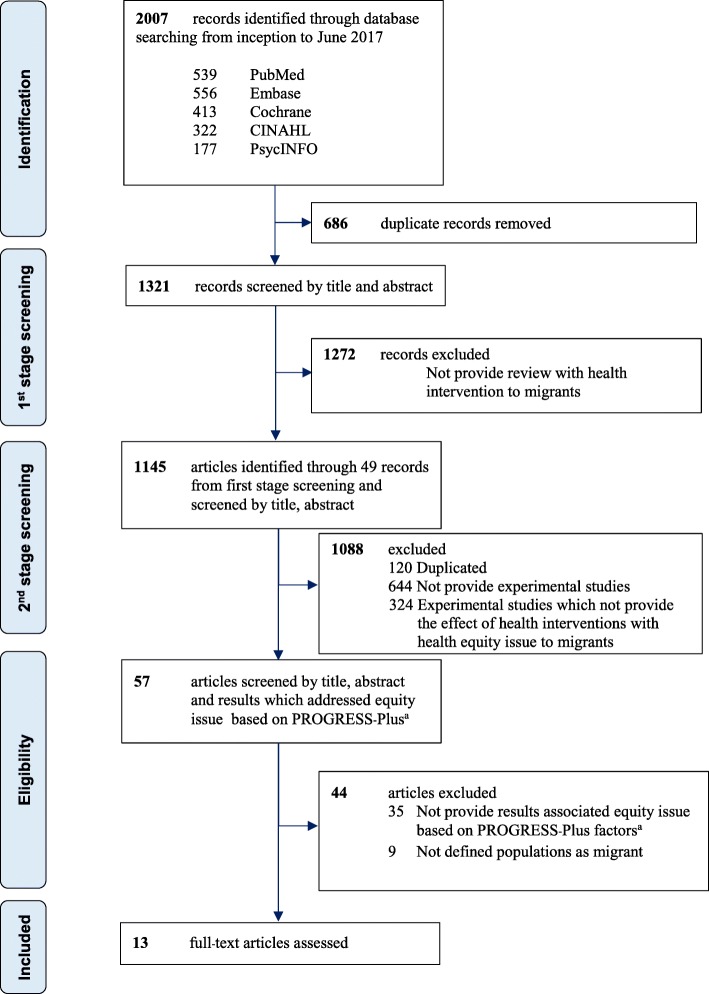
PRISMA Flow Diagram of the literature search and selection process. ^a^Data on health equity: PROGRESS-Plus - Place of residence, Race/ethnicity/culture/language, Occupation, Gender/sex, Religion, Education, Socioeconomic status, Social capital and “Plus” to indicate other possible factors such as disease status or disability

We identified 2007 records, in which 49 reviews met the first-stage inclusion criteria. Of these, 1145 primary studies were screened to identify experimental studies which addressed health equity of migrants, using one or more PROGRESS-Plus factors. Based on the title and abstract screening, 120 and 644 were excluded because they were duplicates and non-experimental study designs, respectively. Out of the remaining 381 experimental studies, 324 did not include health equity attributes, leaving 57 studies for full-text retrieval. A total of 13 studies was included in this review after review at full text stage for eligibility. The flow of the included studies in this review is shown in Fig. 1.

### Study characteristics

Table 3 shows the summary of results extracted from the included articles. Studies included in this review were conducted in the United States (US) (*N* = 11) [22–32], Spain (*N* = 1) [33], and Norway (*N* = 1) [34]. The publication year ranged from 2000 to 2015. We included eight randomized controlled studies (RCT) and five quasi-experimental studies.

**Table 3:** Characteristics and main findings of studies included in this review

Author, year	Study design	Inter-vention type^a^	Intervention details	Migrants		PROGRESS-PLUS factor^b^	Outcomes	Findings
				Origin	Host country			
Bastini R 2015	Cluster-randomized trial	2	Church-based intervention; single-session small-group discussion supplemented by print materials (Intervention vs Control group)	Korean	US		HBV testing receipt at 6-month follow up	Overall, the intervention produced a statistically significant intervention effect [OR 4.9, *P* < 0.001; 95% CI 2.4–9.9], with 19% of intervention and 6% of control group participants reporting receipt of HBV serologic test at the 6-month follow up
						R - Religion		Statistically significant intervention effects were observed within small (OR 5.3,1.7–16.5, p 0.004), medium (OR 6.4,2.5–16.3,*p* < 0.001), and non-Koreatown churches (OR 8.6,3.9–19.4,*p* < 0.001), compared to control group
								Randomized participants excluding those from large, Koreatown churches with documented contamination, the overall effect of the intervention remained significant (OR 5.7,3.1–10.3, *p* < 0.001) and statistically significant intervention effects were also observed among large and Koreatown churches.
Braschi CD 2014	RCT	1	Patient navigation (PN) calls prior to the screening colonoscopy procedure. Written bowel preparation instructions were mailed after the scheduling call: [1] Enhanced PN: culturally targeted message emphasizing importance of SC for Latinos and attended to patients’ concerns [2] Standard PN	Latin	US		Screening colonoscopy (SC) completion	Overall: There was no difference in SC completion between PN groups (80.9 and 79.0%).
						R1 - Language acculturation		Logisitic regression: The language acculturation subscale was predictor of colonoscopy completion (*P* < =0.000, OR = 2.223, 95%CI 1.470–3.361)
						S1 - Annual income		Annual income above $10,000 (OR = 1.97, 1.09–3.56, p0.026) was independent predictors of completion, compared with income below $10,000
						S1 - Insurance		Insurance type was not the predictor of completion (OR for private/self-pay 2.54, 0.82–3.68, *p* = 0.11, compared to Medicare/Medicaid)
Chiang CY 2009	Pre/Post Quasi-experimental	2	Culturally modified walking (CMW): 8-week walking program and encouragement from older adult in community or church authority (Intervention vs control group)	Chinese	US	E - Education	Duration of walking	Subjects with lower education walked more than those with higher education (F 4.3, *p* < 0.05) in the intervention group
							Blood pressure	The SBP of subjects with higher education decreased more at posttest than those with lower education (F 5.02, *p* < 0.05) in the intervention group
						R - Religion	Duration of walking	Taoists or Buddhists walked more than those were Christians, including Catholics (F 3.13, *p* < 0.05)
							Blood pressure	No differences among religions
						S - Socioeconomics (State of Change; SOC)	Duration of walking	Duration of walking was significantly different between the preparation and maintenance stages (F 3.97, *p* < 0.05) (support in relation to the main effect of SOC)
Elder JP 2000	Quasi-experimental	1	Incorporating nutritional behavior change materials into English-language curricula	Latin	US			Overall: The intervention and control group changed differentially on total cholesterol: HDL ratio(F3.57,*p* < 0.05), systolic blood pressure(F4.04,*p* < 0.05), fat avoidance(F11.56,*p* < 0.001), nutrition knowledge(F20.67,*p* < 0.001), and stress knowledge (F27.62,*p* < 0.001]
						R - Language (Spanish literacy)	Nutrition knowledge	Nutrition knowledge gain was greater among those with medium and high Spanish literacy than among those with low literacy (Mentioned in the result of study but data are not shown in term of value)
Fang CY 2007	2group Pre/Post Quasi-experimental	1	2-h small-group education session focused on cervical cancer risk factors, prevalence rates, and the benefits of screening and early detection, particularly in relation to the life roles of Asian women e.g., social norms, family responsibilities (Intervention vs control group)	Korean	US		Screening behavior	Screening rates were significantly higher in the intervention group (83%) compared with the control group (22%), ×2 [1] = 41.22, *P* < 0.001
						S1 - Marital status		Multivariate logistic regression: The marital status was not associated with screening uptake (OR 0.78 (0.17–3.49) *p* = 0.74)
						S1 - Insurance		Multivariate logistic regression: The insurance status was significant associated with screening uptake (OR 9.53 (1.30–69.66) *p* = 0.03)
Jandorf L 2008	RCT	2,3	Culturally Specific Educational Program: educate about breast and cervical cancers and the importance of routine screening (Intervention vs control group)	Latin	US	S1 - Marital status	Clinical Breast Examination (CBE)	Women who were married or living with partners were significantly MORE LIKELY to be adherent for CBE (OR 2.0, 1.1.-3.7, *p* = 0.0303) at the follow-up as compared with those who were not
							Breast Self-Examination (BSE)	No different BSE screening at follow-up among marital status
							Mammogram	No different Mammography at follow-up among marital status
							Pap smear	No different Pap test at follow-up among marital status
								Overall: Screening rates were significantly higher for the intervention versus the control group for: CBE; 48% vs. 31%; adjusted OR 2.2 (1.1–4.2), BSE (45% vs. 27%; aOR 2.3; 1.1–5.0), and Pap testing (51% vs. 30%; aOR 3.9; 1.1–14.1), but not for mammography (67% vs. 58%; aOR 0.7; 0.1–3.6)
Jimenez-Fuentes MA 2013	RCT	1	two approaches for the treatment of latent tuberculosis infection (LTBI): 6 months of isoniazid (6H) vs. 3 months of isoniazid plus rifampicin (3RH).	Eastern Europe/ South and central America/ Africa/ Asia	Spain	E - Education	non-adherence to preventive chemotherapy of TB	Variables associated with non- adherencewere diagnosis by illegal immigration status (OR 1.48,95%CI 1.01–2.15, *P* = 0.03), unemployment (OR 1.91,95%CI 1.28–2.85, *P* = 0.0008), illiteracy (OR 1.73,95%CI 1.04–2.88, *P* = 0.02), lack of family support (OR 3.7, 95%CI 2.54–5.4, *P* = 0.001)
						S - Immigration status		
						S - Labor status		
						S - Family status		
						G - Gender		Gender was not associated with non- adherence (OR 1.4, 0.77–1.69, p 0.49, compared male to female)
								Overall: the rate of adherence was greater in the 3RH than in the 6H arm (72% vs. 52.4%, *P* = 0.001)
Kagawa-Singer M 2009	Quasi-experimental	2	Culturally informed educational program: education sessions with video, games, flipchart about importance and step of breast cancer screening (Intervention city vs Non-intervention city)	Hmong	US	E - Education	Breast Self-Examination (BSE)	subgroup analysis: BSE screening receipt increased in participants with No schooling in US in the intervention group with OR 4.32 (1.05, 17.71) (*p* < 0.05) compared with control group
							Clinical Breast Examination (CBE)	No difference in CBE receipt among education in US between 2 groups
							Mammogram	No difference in mammogram among education in US between 2 groups
								Overall: The intervention group significantly predicted increases in all 3 breast cancer screenings after controll for years in US, age, marital status, language, years of education, and health insurance status (OR for BSE 20.06,3.08–130.79,*p* < 0.001; OR for CBE 12.16,1.44–102.74,*p* < 0.05; OR for mammogram 6.75,1.55–29.39,*p* < 0.01)
Mishra SI 2007	RCT	2	Breast Cancer Education Program: booklets; skill building and behavioral exercises; and interactive group discussionsessions	Samoan	US	P – Place of origin	Mammogram receipt	No differences mammogram receipt among country of birth
						S - Marital status		Marital status with current married increased self-reported receipt of mammogram compared with currently single status with OR 1.31 (1.01, 1.70) *p* = 0.041
						S - Employment status		Employed status increased self-reported receipt of mammogram compared with unemployed status with OR 1.48 (1.15, 1.13) *p* = 0.005
						E - Education		No differences mammogram receipt among education level
						S - Insurance status		No differences mammogram receipt among insurance status
						S – Family income		Annual family income ≥ $20,000 increased self-reported receipt of mammogram compared with income under $10,000 with OR 1.53 (1.10, 2.12) *p* = 0.012
						R – Language of interview		No differences mammogram receipt among language of interview with Samoan compared to English
						’PLUS’ Others - Age		No differences mammogram receipt among age group
								Overall, there was no statistically significant intervention effect with OR 1.26 (0.74–2.14) *p* = 0.39 compared with control group
Nguyen TT 2009	RCT	3	Compare Lay health workers and media education program (LHW + ME) with Media education (ME): group session with flip chart andbooklet as the basis for factual information and for motivation, 2 phone calls with in 1–2 month to explain and using media education via TV & radio advertisements, newspaper advertisements & articles	Vietnamese	US	–	Mammogram	The LHW + ME group increased receipt of mammography ever and mammography in the past 2 years (84.1 to 91.6% and 64.7 to 82.1%, p 0.001) while the ME group did not
						–		Overall: after controlling for LHW agency, baseline mammogram receipt status, age, English proficiency, years in the U.S., education, employment, marital status, family history of breast cancer, household clusters, and health insurance with OR 3.62 (1.35–9.76)
						S1 - Insurance		Multivariate analysis: Participants with Health insurance increased mammogram receipt within 2 years compared with no insurance with OR 2.84 (1.73, 4.69)
						Others - Age		Multivariate analysis: Participants with 40–49 year of age decreased mammogram receipt within 2 years compared with 50–64 year of age with OR 0.51 (0.30, 0.87)
						–	Clinical Breast Examination (CBE)	The rate for ever having had CBE increased in both the ME and LHW + ME groups, with the LHW + ME group having a significantly greater increase (17.1% vs 5.9%, *p* < 0.01). Similarly, receipt of a CBE within the past 2 years increased in both groups, with the LHW + ME group having a significantly greater increase (23.1% vs 4.2%,*p* < 0.001).
						–		The intervention group OR for ever having had a CBE was 2.94 (1.63–5.30) and for having had a CBE within the past 2 years was 3.04 (2.11–4.37) compared with control (ME) group
						S1 - Insurance		Multivariate analysis: No differences in CBE receipt within 2 years among participant with or without insurance
						Others - Age		Multivariate analysis: Participants with ≥65 year of age decreased CBE receipt within 2 years compared with 50–64 year of age with OR 0.51 (0.31, 0.83)
Raberg Kjollesdal MK 2011	RCT	1,2	Group sessions with culturally adapted materials and discussion: focused on the importance of diet and physical activity for blood glucose regulation (Intervention vs control group)	Pakistan	Norway	E - Education	Food perceptions in terms of health	Changes in perceptions in the intervention group were not significantly related to age,number of years in Norway, years of education or commandof Norwegian language, with the exception that those with higher education have changed the perception of legumes as good for the body (OR 1.13,*p* = 0.01)
Taylor VM 2011	RCT	2	Classes (3 h/sesssion) in English as a second language (ESL) curriculum addressing HBV (Intervention vs control group)	Asian (China/India/Iran/Others)	US		Hepatitis B knowledge scores	Mean scores 3.68 (SD 1.12) among experimental group and 2.87 (SD 1.38) among control group (*P* < 0.001) and remained highly significant (*P* < 0.001) after adjustment for other variables.
						R - Country of origin		Mean scores were higher among experimental group from China, India, Iran, and other Asian countries than their control group counterparts, and the differences between the 2 groups were significant (*P* < 0.05) for China and other Asian countries
Wang X 2010	Quasi-experimental	2,3	Community-based pilot intervention that combined cervical cancer education with patient navigation on cervical cancer screening behaviors	Chinese	US	–	Cervical-cancer screening rate (at 12 month follow-up)	Overall, Screening rates were significantly higher in the intervention group (70%) compared to the control group (11.1%), *p* < 0.001
						R - Language (English proficiency)		Women with poorer English fluency were less likely to obtain screening (OR 0.30, 0.10–0.89, *p* < 0.05), compared to English fluency
						S1 - Insurance		Women who did not have health insurance were less likely to obtain screening (OR 0.15, 0.02–0.96, *p* < 0.05), compared to women with health insurance
						Others - Age		12-month screening behavior was associated with older age (OR 1.08,1.01–1.15, *p* < 0.05)

^a^Type of intervention: 1-Individual directed, 2-Community education, 3-Peer navigator-related, 4-Access-enhancing

^b^Data on health equity: PROGRESS-Plus - Place of residence, Race/ethnicity/culture/language, Occupation, Gender/sex, Religion, Education, Socioeconomic status, Social capital and “Plus” to indicate other possible factors such as disease status or disability

**Fig. 2:**
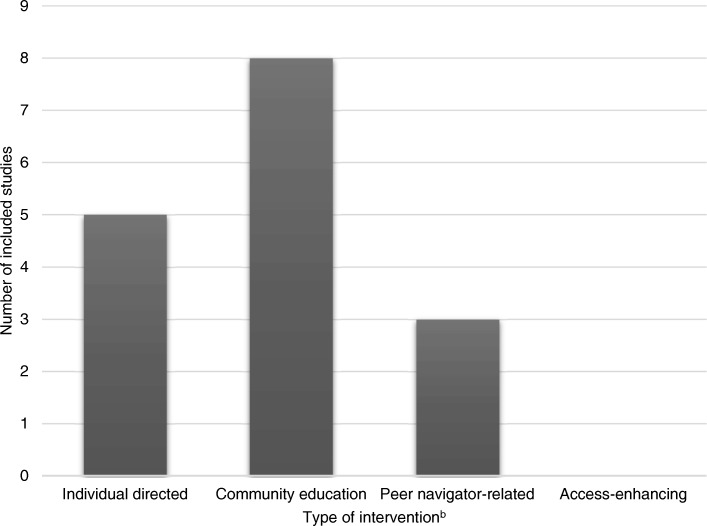
Studies reported the type of intervention in this review^a^. ^a^Type of intervention- Individual directed intervention: to provide information to individual on benefits of screening or intervention; Community education: to provide the intervention through small group workshops or seminars usually conducted by healthcare professionals or trained staffs in the setting; Peer navigator-related intervention: the method by the peer leaders or lay health workers in the community to provide necessary support, follow-up, or referral to help migrants to receive intervention Access-enhancing: to promote screening by reducing financial or linguistic barriers that hamper access to screening services. ^b^ Some studies reported more than one type

Following the study classification system used in the scoping review in migrant populations [6, 35], these 13 studies were categorized as individual directed (*N* = 5) [24, 25, 31, 33, 34], community education (*N* = 8) [22, 23, 26–29, 32, 34] and peer-navigator related intervention (*N* = 3) [26, 30, 32] (Fig. 2). The individual directed interventions aimed to provide the information on the benefits of a screening program or health intervention. Examples included the use of patient navigation by calling individuals prior to a screening colonoscopy procedure, mailing the instructions for bowel preparation after the scheduling call, and emphasizing the importance of the screening for the Latin migrants in the US [24]. Other individual directed interventions were the incorporation of nutritional behaviour change instruction into English-language curriculum for the Latin migrants in the US [25], the provision of an education session about the importance of diet and physical activity for blood glucose regulation for Pakistan migrants in Norway [34] or cervical cancer risk factors, prevalence rates, and the benefits of screening and early detection for Korean people [31] in the US, and the treatment of latent tuberculosis infection with 6 months of isoniazid or 3 months of isoniazid plus rifampicin for migrants from Eastern Europe, South and Central America, Africa, Asia in Spain [33]. The second category of intervention, community education, consisted of small group workshops conducted by the healthcare professionals or staff. They included a small-group discussion on hepatitis B virus testing for Korean and other Asian migrants in the US [23, 27], a walking program and encouragement from older adults in the community or church authority for Chinese migrants in the US [28], the importance of breast or cervical cancer routine screening for Samoan [22], Chinese [26], Hmong [29], and Latin [32] migrants in the US, and the importance of diet and physical activity for Pakistan migrants in Norway [34]. The last group of interventions were peer navigator-related interventions which provided necessary support, follow-up, or referral methods by leaders or lay health workers in the community to help migrants to receive the intervention. Examples included the provision of the information on the importance of breast or cervical cancer and screening program by community staff for the Chinese [26] and Latin [32] migrants in the US, the use of media education and telephone communication provided by lay health workers to provide information about breast cancer screening for Vietnamese migrants in the US [30].

### Quality assessment

Six of 8 RCT studies were rated as a high potential risk of bias due to bias in deviations from intended interventions and bias in outcome measurement. The quality assessment for randomized trials included in this review is shown in Table 4. Quality assessment for quasi-experimental studies were found to be moderate and serious risk of bias for 4 and 1 studies, respectively. Further detail on quality assessment for quasi-experimental studies included in this review is presented in Table 5.

**Table 4:** Quality assessment for randomized controlled trials included in this review

Study	Domain					Overall risk of bias
	1. Randomization process	2. Deviation from intended interventions	3. Missing outcome data	4. Measurement of outcome	5.Selection of the reported results	
Bastani 2015	Some concerns^a,b^	Low risk	Low risk	High risk	Low risk	High risk
Braschi 2014	Some concerns^b,c^	Low risk	Low risk	Low risk	Low risk	Some concerns
Jandorf 2008	Some concerns^a,b^	Some concerns	Low risk	Low risk	Low risk	Some concerns
Jimenez-Fuentes MA 2013	High risk	High risk	Some concerns	Some concerns	Some concerns	High risk
Mishra, 2007	High risk	High risk	Low risk	High risk	Low risk	High risk
Nguyen, 2009	Some concerns^a,b^	Some concerns	Low risk	High risk	Low risk	High risk
Raberg Kjollesdal MK 2011	Low risk	Some concerns	Some concerns	Low risk	Low risk	High risk
Taylor VM 2011	Some concerns^a,b^	Some concerns	Low risk	High risk	Low risk	High risk

^a^No information was provided about allocation sequence

^b^No information was provided about allocation concealment

^c^No information was provided about baseline imbalance

**Table 5:** Quality assessment for quasi-experimental studies included in this review

Study	Domain							Overall risk of bias
	1. confounding	2. selection of participants into the study	3. classification of intervention	4. deviations of intended interventions	5. missing data	6. measurement of outcomes	7.selection of the reported results	
Chiang 2009	Moderate risk	Moderate Risk	Low risk	Low risk	Low risk	Low risk	Low risk	Moderate Risk
Elder 2000	Moderate risk	Low risk	Low risk	Low risk	Low risk	Low risk	Low risk	Moderate risk
Fang 2007	Moderate risk	Low risk	Low risk	Low Risk	Low risk	Moderate Risk	Low risk	Moderate Risk
Kagawa-Singer, 2009	Moderate risk	NI	Low risk	NI	Low risk	Serious risk	Low risk	Serious risk
Wang X 2010	Moderate risk	Moderate risk	Low risk	Low risk	Low risk	Moderate risk	Low risk	Moderate risk

*NI* No information, *NA* Not applicable

### Assessing evidence of inequity based on PROGRESS-Plus

**Fig. 3:**
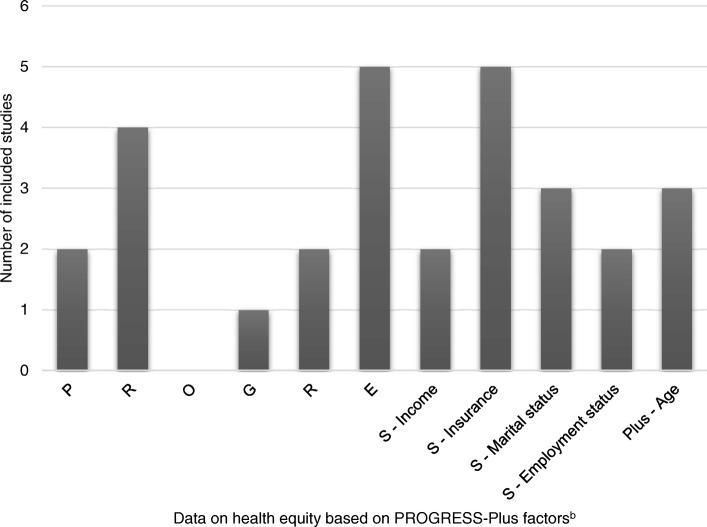
Studies reported the potential difference of the effect of intervention on outcome-based PROGRESS-Plus^a^ in this review. ^a^Data on health equity: PROGRESS-Plus - Place of residence, Race/ethnicity/culture/language, Occupation, Gender/sex, Religion, Education, Socioeconomic status, Social capital and “Plus” to indicate other possible factors such as disease status or disability. ^b^Some studies reported more than one factor

We found none of the included studies reported the effect of the intervention on reducing inequity according to PROGRESS-Plus. However, all included studies reported the potential difference of the effect of the intervention on outcomes based on PROGRESS-Plus. In addition, six out of 13 studies explicitly discussed the effect of the intervention on equity attributes based on PROGRESS-Plus factors [24, 26, 27, 30–32]. The determinants included in the studies were: place of residence (*N* = 2) [22, 23], language (*N* = 4) [22, 24–26], gender (*N* = 1) [33], religion (*N* = 2) [27, 28], education (*N* = 5) [22, 28, 29, 33, 34], socioeconomic status (*N* = 9) [22, 24, 26, 28–33], and age (*N* = 3) [22, 26, 30]. No study examined the difference between occupation and social capital. The frequency of reporting across PROGRESS-Plus among included studies is presented in Fig. 3.

### Place of residence

Two studies [22, 23] looked at the association between country of birth and the outcome of an educational program. One RCT [22] focusing on the breast cancer education program among Samoan migrants in the US reported no significant association between country of birth in American Samoa, an unincorporated territory of the United States, compared to other areas of Samoa (OR 1.19, 95% CI; 0.82–1.74, p 0.365). Another RCT [23] showed the association between country of birth and hepatitis B knowledge score among Asian migrants in the US who participated in classes in English as a second language curriculum which provided hepatitis B virus information. Mean scores were higher among the experimental group than the control group, especially the mean score between groups was significant (*p* < 0.05) for China [3.56 (SD 1.14) vs 2.94 (SD 1.25), p 0.02] and other Asian countries (Afghanistan, Cambodia, Hong Kong, Korea, Taiwan or Vietnam) groups [3.82 (SD 0.95) vs 2.44 (SD 1.46), p 0.002].

### Race/ethnicity/culture/ language

Two RCTs [22, 24] and two quasi-experimental studies [25, 26] examined the association between language proficiency and the outcome of the intervention. The two RCTs were conducted in the US. One RCT [22] focusing on a breast cancer educational program among Samoan migrants in the US showed that there was no difference in mammogram receipt between interview languages (Samoan vs English, OR 0.75; 95% CI; 0.52, 1.06, *p* < 0.106). Another RCT [24] examined the effect of patient navigation by calling prior to a screening colonoscopy procedure and mailing bowel preparation among Latin migrants. The multiple regression analysis revealed that the language acculturation subscale in Latin migrants was a significant predictor of colonoscopy completion (OR 2.223; 95% CI; 1.470–3.361, *p* < 0.001).

One quasi-experimental study [25] assessed the effect of incorporating nutritional behaviour change materials into curricula among Latin migrants in the US. The authors mentioned that nutrition knowledge gain was greater among migrants with medium and high Spanish literacy than those with low literacy. Another study [26] assessed the impact of a community-based intervention that combined cervical cancer education with patient navigation for Chinese migrants in the US and revealed that women with poorer English proficiency were less likely to obtain cervical cancer screening at 12-month follow up compared to those with higher English fluency (OR 0.30; 95% CI; 0.10–0.89, *p* < 0.05).

### Gender

We found one RCT [33] examining the difference in non-adherence to preventive chemotherapy of latent tuberculosis infection between males and females among migrant populations (including Eastern Europe, South and Central America, Africa and Asia) in Spain. Gender was not associated with non-adherence when comparing male to female migrants (OR 1.4; 95%CI; 0.77–1.69, p 0.49).

### Religion

We found one RCT [27] and one quasi-experimental study [28] assessing the association between religion and the outcome. The RCT [27] was conducted in the US to evaluate effectiveness of a church-based intervention with small group discussion supplemented by materials about hepatitis B screening among Korean migrants to improve Hepatitis B virus testing at 6-month follow up. Statistically significant intervention effects were observed within small (OR 5.3; 95%CI; 1.7–16.5, p 0.004), medium (OR 6.4; 95%CI; 2.5–16.3, *p* < 0.001), and non-Korean town churches (OR 8.6; 95%CI 3.9–19.4, *p* < 0.001), compared to the control group. A pre-post quasi-experimental study [28] aimed to assess the effect of culturally modified walking with encouragement from older adults in the community among Chinese migrants in the US. The results showed that Taoists or Buddhists spent more time walking than Christians, including Catholics (*p* < 0.05) but no difference in blood pressure was observed across religious groups.

### Education

Two quasi-experimental studies [28, 29] and three RCTs [22, 33, 34] looked at the association between education level and health outcomes. The first quasi-experimental study [28] was a culturally modified walking program among Chinese migrants in the US. The study showed that participants with middle school or lower education walked more than those with higher education (*p* < 0.05). However, it was found that the decrease in systolic blood pressure was much larger in those with higher education than those with lower education (*p* < 0.05) at post-test in the intervention group. The second quasi-experimental study [29] assessed the effect of an education program about the importance and steps of breast cancer screening among Hmong migrants in the US. The subgroup analysis revealed that breast self-examination screening increased in participants with no schooling in the intervention group compared with the control group (OR 4.32; 95%CI; 1.05–17.71, *p* < 0.05). However, no difference in clinical breast examination and mammography receipt between groups was detected. One RCT [22] provided a breast cancer educational program among Samoan migrants in the US. The result showed there was no difference in mammogram receipt across education level when comparing women with more than 12 years of education (OR 1.55; 95%CI; 0.98–2.45, p 0.063) and women with 9–12 years of education (OR 1.19, 95%CI; 0.88–1.60, p 0.259) to women with equal or less than 8 years of education. Another RCT [34] examined the effect of a group session using culturally adapted materials with a discussion panel focusing on the importance of diet and physical activity for blood glucose regulation among Pakistan migrants in Norway. The results revealed that changes in the perceptions in the intervention group were not significantly related to age, number of years in Norway, years of education or command of the Norwegian language, with the exception that those with higher education had changed their perception of legumes as good for the body (OR 1.13, p 0.01). The third RCT [33] reported the effect of a treatment of latent tuberculosis infection among migrant populations in Spain. The univariate analysis indicated that illiteracy was associated with non-adherence to drug treatment (OR 1.73; 95%CI; 1.04–2.88, p 0.02).

### Socio-economic status

#### Income

One RCT [24] examined the effect of patient navigation intervention among Latin migrants in the US. The study found that those with higher income levels had greater uptake of preventive service. In particular, an annual income above $10,000 was an independent predictor of the completion of colonoscopy screening from patient navigation program compared to those with income below $10,000 (OR 1.97, 95%CI; 1.09–3.56, p 0.026).

#### Insurance status

Three RCTs [22, 24, 30] and two quasi-experimental studies [26, 31] evaluated the association between insurance status and their health outcomes. The first RCT [24] showed that insurance status was not associated with completion of colonoscopy screening (OR 2.54; 95%CI; 0.82–3.68, p 0.11) when comparing the private/self-pay insurance group to the Medicare/Medicaid scheme group among Latin migrants in the US who received the intervention. The second RCT [22] mentioned that there was no difference in mammogram receipt between insurance status (OR 1.21; 95%CI; 0.92–1.97, p 0.125) in the intervention group among Samoan migrants in the US who attended the breast cancer educational program. However, the third RCT [30] conducted a program by using lay health workers with media education about cervical cancer screening among Vietnamese migrants in the US. The study revealed that participants with health insurance increased mammogram receipt within 2 years compared to the group with no insurance (OR 2.84; 95%CI; 1.73–4.69) Moreover, two quasi-experimental studies [26, 31] revealed that insurance status was significantly associated with completion of the screening program. One quasi-experimental study [31] provided small group educational sessions focusing on cervical cancer and the benefits of screening and early detection among Korean migrants in the US. The multiple logistic regression demonstrated that insurance status was significantly associated with cervical cancer screening uptake (OR 9.53; 95%CI; 1.30–69.66, p 0.03). Another study [26] evaluated a community-based intervention which combined cervical cancer education with patient navigation to increase the receipt of a screening program among Chinese migrants in the US. Women without health insurance were less likely to obtain the screening program, compared to women with health insurance (OR 0.15; 95%CI; 0.02–0.96, *p* < 0.05).

#### Marital status

One quasi-experimental study [31] examined the effect of a culturally modified walking program among Korean migrants in the US and revealed that marital status was not associated with screening uptake for cervical cancer (OR 0.78; 95%CI; 0.17–3.49, p 0.74). However, two RCTs [22, 32] showed that women who were married were significantly more adherent to screening uptake for breast cancer. One RCT [32] provided an educational session of cervical and breast cancer among Latin migrants in the US. This study demonstrated that women who were married or living with partners were significantly more likely to have had a clinical breast examination (OR 2.0; 95%CI; 1.1–3.7, p 0.03). In addition, another RCT [22] assessed a breast cancer education program among Samoan in the US. Mammogram receipt with current married status was significantly higher than those who were single (OR 1.31; 95%CI; 1.01–1.70, p 0.041).

#### Employment status

Two RCTs [22, 33] showed that unemployment status might lead to poor outcomes. One RCT [33] reported that unemployment status was associated with non-adherence to drug treatment for latent tuberculosis among migrants in Spain (OR 1.91; 95%CI; 1.01–2.15, p 0.03). Another RCT [22] provided a breast cancer educational program and demonstrated that those who were currently employed increased self-reported receipt of mammogram among Samoan migrants in the US, compared to those who were unemployed (OR 1.48; 95%CI; 1.15–1.13, p 0.005).

#### Others

In addition to PROGRESS as determinants of health intervention effects, three studies [22, 26, 30] examined the age of migrants as ‘plus’ or other determinants of health equity. Two RCTs [22, 30] and one quasi-experimental study [26] examined the association between age and outcomes.

One RCT [22] evaluated the effect of breast cancer education program among Samoan migrants in the US. The study found no difference in mammogram receipt among age groups when comparing participants aged 42–49 years (OR 1.21; 95%CI; 0.82–1.80, p 0.337) with those aged 50–64 years (OR 1.29; 95%CI; 0.90–1.86, p 0.171) to participants aged 65 years or older, respectively. Another RCT [30] examined the effect of lay health workers with media education for breast cancer screening among Vietnamese migrants in the US. The multivariate analysis demonstrated that the participants aged 40–49 years had fewer mammograms within 2 years, compared to those aged 50–64 years (OR 0.51, 95%CI; 0.30–0.87). In contrast, the participants aged 65 years or older had fewer clinical breast examinations within 2 years compared to those aged 50–64 years (OR 0.51, 95%CI; 0.31–0.81).

One quasi-experimental study [26] showed that the outcomes in the 12-month interval following a community-based program among Chinese migrants in the US, cervical screening rate were significantly higher in the intervention group (70%) compared to the control group (11.1%). Hierarchical logistic regression analysis indicated that screening behavior was associated with older age (OR 1.08, 95% CI; 1.01–1.15, *p* < 0.05).

## Discussion

We systematically identified experimental studies that evaluated the effects of interventions on health outcomes of migrant populations and assessed whether equity was addressed in the published literature based on PROGRESS-Plus factors. None of the included studies examined the effect of health interventions on reducing inequity amongst migrant populations. However, some studies reported the potential difference of the effect of the intervention on outcomes based on PROGRESS-Plus. Our findings suggest for the need to develop interventions to improve health outcomes of migrants and incorporate the equity attributes as part of outcome measurement, to support the goal to achieve SDG on the reduction of inequalities [1, 5].

Our results are consistent with the findings from previously published reviews on interventions related to vaccination [36] and health care models among migrant populations [37]. Both reviews [36, 37] similarly mentioned that none of the included studies reported the effectiveness or measured the impact of interventions on health inequity in the populations. In terms of inclusion of equity attributes in the studies, only 3.41% in our review (13 out of 381 experimental studies) reported variation in the outcomes by equity attributes. Less than half of these discussed equity issues in their discussion and conclusions. This is similar to what was reported in a previous review [36] which reported no increasing trend of consideration of equity attributes in studies over time. These findings highlight a lack of research interest in measuring the effects of interventions in relation to equity attributes among migrant populations. Since addressing equity as part of outcome measurement is an important part of meeting the SDGs; more research on health interventions for migrants incorporating equity attributes based on PROGRESS-Plus factors is strongly encouraged in the future.

The review approach adopted in our review is somewhat different from those in previous reviews. Since we wanted to look at how primary studies on health interventions for migrants have captured equity attributes, we needed to determine a feasible approach that would enable us to identify primary studies. Given the extremely large number of individual studies identified from searching, we chose to use a two-stage systematic review approach. The unit of analysis in our review was at the individual study, while at the review level for previous reviews. We extracted information from primary studies to evaluate the effect of health interventions based on the equity attributes while the previous study [36] performed an overview of systematic reviews. We also included only experimental studies while the previous study [36] included both experimental and observational studies and another study [37] included qualitative research and policy documents.

Several limitations inherent in our review deserve discussion. First, all included studies were conducted in high-income countries including the US, Spain, and Norway. Therefore, our findings of the lack of minimal measurement of equity attributes might have limited generalizability to those studies conducted in low and middle-income countries. Second, six out of 8 included RCTs potentially had a high risk of methodological bias. There remains a need for further improvement of the methodology used in studies assessing the effect of interventions among migrant populations.

## Conclusion

This systematic review has critically highlighted the current health intervention studies targeted at migrants. A paucity of such studies indicates that equity has not been receiving attention and greater attention to equity-focused outcome assessment is needed. To reduce health inequities among these populations, the framework which includes equity attributes based PROGRESS-Plus factors should be incorporated into future implementation research.

## Additional file

Additional file 1: **Table S1.** Search strategy (from inception until June 2017). (DOCX 28 kb)

## Abbreviations

OR: Odds ratio
PROGRESS-Plus: Place of residence, race/ethnicity, occupation, gender, religion, education, socio-economic status, social capital and others
RCT: Randomized controlled trial
SD: Standard deviation
SDGs: Sustainable Development Goals
UN: The United Nations
US: The United States
WHO: World Health Organization
