# Supplementary material for: Assessing evidence of interventions addressing inequity among migrant populations: a two-stage systematic review
Source: Int J Equity Health. 2019 May 6;18:64. doi: 10.1186/s12939-019-0970-x (PMC6501336; doi:10.1186/s12939-019-0970-x)
Supplement: Supplementary file 1 — Table S1. Search strategy (from inception until June 2017). (DOCX 28 kb) [file 12939_2019_970_MOESM1_ESM.docx]

**Additinal file 1: Table S1: Search strategy (from inception until June 2017)**

| **Database** | **Search** | **Query** | **Items found** |
| --- | --- | --- | --- |
| PubMed | #1 | "data synthesis" OR "evidence synthesis" OR metasynthesis OR meta-synthesis OR "narrative synthesis" OR "qualitative synthesis" OR "quantitative synthesis" OR "realist synthesis" OR "research synthesis" OR "synthesis of evidence" OR "thematic synthesis" OR metaanaly* OR meta-analy* OR "scoping stud*" OR meta-ethnograph* OR meta-epidemiological OR “systematic review” OR “scoping review” OR “rapid review” | 199400 |
|  | #2 | refugees OR refugee OR refugee camps OR camp OR refugee OR camps | 101205 |
|  | #3 | aliens OR alien OR emigrants OR emigrant OR foreigners OR foreigner OR immigrants OR immigrant OR migrant OR migrants | 46934 |
|  | #4 | asylum-seekers | 10117 |
|  | #5 | “internally displaced person” | 17 |
|  | #6 | Health | 3746887 |
|  | #7 | (#2 OR #3 OR #4 OR #5) | 145366 |
|  | #8 | #1 AND #6 AND #7 | 539 |
| EMBASE  via Ovid | #1 | "data synthesis" OR "evidence synthesis" OR metasynthesis OR meta-synthesis OR "narrative synthesis" OR "qualitative synthesis" OR "quantitative synthesis" OR "realist synthesis" OR "research synthesis" OR "synthesis of evidence" OR "thematic synthesis" OR metaanaly* OR meta-analy* OR "scoping stud*" OR meta-ethnograph* OR meta-epidemiological OR “systematic review” OR “scoping review” OR “rapid review” | 324194 |
|  | #2 | refugees OR refugee OR refugee camps OR camp OR refugee OR camps | 107010 |
|  | #3 | aliens OR alien OR emigrants OR emigrant OR foreigners OR foreigner OR immigrants OR immigrant OR migrant OR migrants | 49191 |
|  | #4 | asylum-seekers | 1216 |
|  | #5 | internally displaced person | 34 |
|  | #6 | Health | 3324592 |
|  | #7 | (#2 OR #3 OR #4 OR #5) | 153494 |
|  | #8 | #1 AND #6 AND #7 | 556 |
| Cochrane | #1 | "data synthesis" OR "evidence synthesis" OR metasynthesis OR meta-synthesis OR "narrative synthesis" OR "qualitative synthesis" OR "quantitative synthesis" OR "realist synthesis" OR "research synthesis" OR "synthesis of evidence" OR "thematic synthesis" OR metaanaly* OR meta-analy* OR "scoping stud*" OR meta-ethnograph* OR meta-epidemiological OR “systematic review” OR “scoping review” OR “rapid review” | 62357 |
|  | #2 | refugees OR refugee OR refugee camps OR camp OR refugee OR camps | 1524 |
|  | #3 | aliens OR alien OR emigrants OR emigrant OR foreigners OR foreigner OR immigrants OR immigrant OR migrant OR migrants | 884 |
|  | #4 | asylum-seekers | 25 |
|  | #5 | “internally displaced person” | 0 |
|  | #6 | Health | 206878 |
|  | #7 | (#2 OR #3 OR #4 OR #5) | 2347 |
|  | #8 | #1 AND #6 AND #7 | 413 |
| PsycINFO | #1 | "data synthesis" OR "evidence synthesis" OR metasynthesis OR meta-synthesis OR "narrative synthesis" OR "qualitative synthesis" OR "quantitative synthesis" OR "realist synthesis" OR "research synthesis" OR "synthesis of evidence" OR "thematic synthesis" OR metaanaly* OR meta-analy* OR "scoping stud*" OR meta-ethnograph* OR meta-epidemiological OR “systematic review” OR “scoping review” OR “rapid review” | 43294 |
|  | #2 | refugees OR refugee OR refugee camps OR camp OR refugee OR camps | 16389 |
|  | #3 | aliens OR alien OR emigrants OR emigrant OR foreigners OR foreigner OR immigrants OR immigrant OR migrant OR migrants | 32511 |
|  | #4 | asylum-seekers | 926 |
|  | #5 | internally displaced person | 11 |
|  | #6 | Health | 574404 |
|  | #7 | (#2 OR #3 OR #4 OR #5) | 47608 |
|  | #8 | #1 AND #6 AND #7 | 177 |
| CINAHL | S1 | "data synthesis" OR "evidence synthesis" OR metasynthesis OR meta-synthesis OR "narrative synthesis" OR "qualitative synthesis" OR "quantitative synthesis" OR "realist synthesis" OR "research synthesis" OR "synthesis of evidence" OR "thematic synthesis" OR metaanaly* OR meta-analy* OR "scoping stud*" OR meta-ethnograph* OR meta-epidemiological OR “systematic review” OR “scoping review” OR “rapid review” | 95949 |
|  | S2 | refugees OR refugee OR refugee camps OR camp OR refugee OR camps | 10516 |
|  | S3 | aliens OR alien OR emigrants OR emigrant OR foreigners OR foreigner OR immigrants OR immigrant OR migrant OR migrants | 18863 |
|  | S4 | asylum-seekers | 712 |
|  | S5 | “internally displaced person” | 3 |
|  | S6 | Health | 1262159 |
|  | S7 | (S2 OR S3 OR S4 OR S5) | 28115 |
|  | S8 | S1 AND S6 AND S7 | 322 |
